# Metacognitive Myopia in Hidden-Profile Tasks: The Failure to Control for Repetition Biases

**DOI:** 10.3389/fpsyg.2018.00903

**Published:** 2018-06-05

**Authors:** Klaus Fiedler, Joscha Hofferbert, Franz Wöllert

**Affiliations:** Department of Psychology, Heidelberg University, Heidelberg, Germany

**Keywords:** hidden profiles, repetition bias, group decision making, meta-cognitive myopia, monitoring and control

## Abstract

The failure to exploit collective wisdom is evident in the conspicuous difficulty to solve hidden-profile tasks. While previous accounts focus on group-dynamics and motivational biases, the present research applies a metacognitive perspective to an ordinary learning approach. Assuming that evaluative learning is sensitive to the frequency with which targets are paired with positive versus negative attributes, selective repetition of targets’ assets and deficits will inevitably bias the resulting evaluations. As selective repetition effects are ubiquitous, metacognitive monitoring and control functions are required to correct for repetition biases. However, three experiments show that metacognitive myopia prevents judges from correction, even when explicitly warned to ignore selective repetition (Experiment 1), when same-speaker repetitions rule out social validation (Experiment 2) and when blatant debriefing enforces superficial corrections (Experiment 3). For a comprehensive understanding of collective judgments and decisions, it is essential to take metacognitive monitoring and control into account.

## Introduction

Democratic societies rely on the belief that arduous tasks that exceed individual persons’ capacity can be managed collectively. Performance and motivation can be enhanced if the overall workload is divided. However, for many judgment and decision problems – such as health risk assessment or personnel selection – the need to coordinate and integrate collective efforts creates a serious difficulty. Information can vary in trustworthiness and validity, arguments may be redundant or in conflict, and individual opinions may rely on different sources and sample sizes. Still, in democratic societies, virtually all important decisions are made collectively.

Despite the trust in the superiority of collective knowledge and in the wisdom of crowds (Surowiecki, 2004; Mannes et al., 2014), several decades of empirical research have drawn a rather pessimistic picture. Collective brainstorming was shown to decrease productivity (Diehl and Stroebe, 1987), group discussion can cause polarization and over-statement (Brauer et al., 1995; McCauley, 1998), and others’ advice is not utilized appropriately (Yaniv et al., 2009).

### Conspicuous Evidence From Hidden-Profile Tasks

Research on hidden profile-tasks illuminates this failure to exploit the potential advantage of collective wisdom (Stasser and Titus, 1985, 2003; Lu et al., 2012; Schulz-Hardt and Mojzisch, 2012). In this paradigm, part of the information about decision options (applicants, products) is shared by everybody, while other, unshared information is exclusively available to single individuals (see **Table 1**). Although Candidate A (six positive and three negative attributes) is clearly superior to Candidate B (three positive and six negative attributes), the subset of information available to all three individual judges J1, J2, and J3 favors B (three positive; two negative) over A (two positive; three negative). This is possible because A’s few deficits and B’s few assets are shared (dark gray) whill agree on a wrong decision. The only chance to uncover the hidden profile seems to be the collective exchange all raw arguments about all candidates’ assets and deficits. However, a growing body of evidence shows that people rarely manage to transcend their individual perspective and to identify a hidden profile (Lu et al., 2012; Schulz-Hardt and Mojzisch, 2012). Several explanations that have been offered for this persistent deficit converge on emphasizing group-dynamic influences and social motives.

**Table 1:** Structure of a hidden-profile problem.

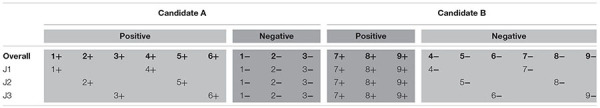

*Natural numbers represent positive(1+… 9+) and negative arguments (1-… 9-). Although the overall information clearly shows that Candidate A is superior to Candidate B, the information available to each of three individual judges (J1,J2, J3) raises a more positive impression of B than A. This is because A’s deficits and B’s assets are shared (dark gray) whereas A’s assets and B’s deficits are unshared (light gray).*

Most prominent accounts focus on a shared-information bias. Shared arguments are more likely to be mentioned and repeated in group discussions than unshared arguments (Stasser et al., 1989; Larson et al., 1994; Mesmer-Magnus and DeChurch, 2009), for two reasons. First, shared arguments are known by more than one discussant and are therefore more likely to be mentioned by at least one discussant than unshared arguments (Larson et al., 1994; Larson and Harmon, 2007). Second, shared arguments are socially rewarding and serve to enhance one’s self-esteem (Wittenbaum et al., 1999). Complementing the shared information bias is a bias to discuss (Dennis, 1996; Faulmüller et al., 2012) or to believe in the validity of preference-consistent arguments (Edwards and Smith, 1996; Greitemeyer and Schulz-Hardt, 2003; Faulmüller et al., 2010). Perceived validity should be enhanced when arguments are shared or consistent with one’s own preferences (Yaniv and Kleinberger, 2000; Volzhanin et al., 2015).

Other accounts have started to examine the cognitive basis of the shared-information bias. As shared arguments are introduced and repeated more frequently (Stasser, 1988; Stasser et al., 1989; Larson and Harmon, 2007), they have a natural memory advantage over unshared arguments. This advantage could interfere with the solution of hidden profile tasks, which draw heavily on the utilization of less well memorized unshared and preference-inconsistent items. Indeed, a number of classical studies testify to the extra persuasive impact of information repetition (Wilson and Miller, 1968; Chalmers, 1971; Cacioppo and Petty, 1979) and to the enhanced attractiveness and preference due to repeated exposure (Zajonc, 1968; Bornstein, 1989). Similar biases favoring repeated arguments can be found in a few hidden-profile studies (Van Swol et al., 2003; Schulz-Hardt et al., 2016).

However, despite the evidence on the advantage of shared or preference-consistent arguments, hidden-profile research has so far not considered an alternative explanation in terms of the simple and uncontested principle that all inductive learning increases with the number of trials. Without any group discussion or prior commitment to individual preferences, and independent of motivational factors such as social utility or subjective validity of arguments, when every item is given the same attention in an unbiased process, evaluative-learning should reflect the number of trials providing positive and negative evidence for different targets. For every stimulus item linking a target to a positive (negative) stimulus item, an increment (decrement) should be added to the evaluation of that target. This valence-updating process should be sensitive to repetitions, not only to novel stimuli, as evident from work on evaluative conditioning (Hofmann et al., 2010) and instance-based learning (Gonzalez and Dutt, 2011). Thus, an unbiased learning mechanism affords a sufficient explanation of the impact of repetition, independent of motivated biases like social sharing, preference consistency, or social validation (Boos et al., 2013).

While such an unbiased, ordinary-learning account calls for the manipulation of repetition as independent variable, almost all previous studies have treated repetition as a dependent variable, showing that shared information is likely to be repeated. Moreover, the two available publications by Van Swol et al. (2003) and by Schulz-Hardt et al. (2016) rely on restricted task set-ups (e.g., including only two-choice alternatives rather than profiles over several targets; convenient protocol sheets reducing memory demands; repetition confounded with preference consistency). Theoretically, both studies focus on distinct cognitive illusions. Van Swol et al. (2003) interpret the obtained repetition bias in terms of a truth bias (Arkes et al., 1991; Boehm, 1994). A similar point is made by Weaver et al. (2007), who argue that the enhanced fluency of repeated arguments should produce a repetition bias, regardless of social validation. Schulz-Hardt et al. (2016) believe in a projective variant of social validation, assuming that repetition leads people to infer that other people share repeated opinions.

### Ordinary Learning and Metacognitive Myopia

The aim of the present article is different from all previous work on hidden profiles. Starting from basic premise that learned evaluations are sensitive to the number of trials, we provide participants with unequal opportunities to learn positive and negative evaluations of four target persons. Impression judgments should reflect the number of trials conveying targets’ assets and deficits. Whether an argument is new or redundant, whether repeated arguments stem from the same or from independent sources, whether learning experience is fluent or effortful, taking place in group discussions or individual encounters, a basic prediction says: evaluation learning is an increasing monotonic function of the frequency of positive minus the negative arguments.

To be sure, amount of information may be reduced when the stimulus series involves repeated, overlapping, or fully redundant arguments. Yet, merely repeating the same stimuli benefits learning. Although novel and surprising stimuli trigger better learning (Rescorla and Wagner, 1972; Sutton and Barto, 1981), a more fundamental rule says that all trials, whether novel or repetitive, will benefit learning. Even plain repetitions foster rehearsal, elaborate encoding, and consolidation and decrease the chances that arguments will be lost, overlooked, or forgotten.^1^ This basic assumption not only accounts for a variety of biases in judgment and decision making (Fiedler, 1996; Fiedler et al., 2002; Lightle et al., 2009). It also offers a new perspective on hidden profiles.

For an experimental demonstration, it is necessary to deprive the hidden-profile task of other influences but repetition. Such a modified set-up appears in **Table 2**; it is the stimulus distribution used in the experiments below. The entire profile of all information about four candidates, A, B, C, D is available to all individual participants, indicating a clear-cut preference order D > C > B > A.^2^ There is no group discussion, no motive to defend one’s predetermined individual preferences, and no distinction of shared and unshared information. However, the selective repetition of part of the arguments creates a conflict between actual set sizes and repetition frequencies of positive and negative attributes. Although B is clearly inferior to D, B’s fewer assets are repeated more often and B’s more deficits are repeated less often than Ds assets and deficits, respectively, making it easier to learn assets and harder to learn deficits in B than in D. Judgments should thus exhibit a bias to favor B over D.

**Table 2:** Two stimulus distributions (Series 1 and Series 2) used to study repetition biases.

	Candidate A		Candidate B		Candidate C		Candidate D	
**Stimulus distributions used for Experiments 1 and 2**		
Series 1	+	-	+	-	+	-	+	-
Effective	2	4	3	3	4	2	6	2
Presented	5	5	7	3	4	6	6	4
*Arguments selected in pretesting*	*64 64*	*28*	*53 53*	*38*	*6*	*79 79 79*	*56*	*5 5*
	*67 67 67*	*41*	*48 48*	*76*	*17*	*35 35 35*	*49*	*80 80*
		*31*	*12 12 12*	*27*	*7*		*22*	
		*77 77*			*61*		*65*	
							*70*	
							*10*	

Series 2	+	-	+	-	+	-	+	-
Effective	2	4	3	3	4	2	6	2
Presented	5	5	7	3	4	6	6	4
*Arguments selected in pretesting*	*22 22*	*35*	*61 61*	*77*	*67*	*31 31 31*	*12*	*27 27*
	*70 70 70*	*80*	*77 77*	*38*	*17*	*28 28 28*	*8*	*76 76*
		*79*	*10 10 10*	*5*	*53*		*65*	
		*41 41*			*64*		*48*	
							*56*	
							*49*	
**Stimulus distribution used for Experiment 3**		
	+	-	+	-	+	-	+	-
Effective	4	8	6	6	8	4	12	4
Presented	10	10	14	6	8	12	12	8

*Due to selective repetition, the resulting presented frequencies of positive (+) and negative attributes (-) diverge from the effective rates of original attributes describing four candidates A, B, C, and D, due to repetition of selected items. Arguments are represented by their pretest numbers*.

In the present set-up, finding the hidden profile of substantial information requires judges to ignore (the repeated) part of the superficially presented information, unlike the common task set-up in which the hidden profile includes additional (unshared) items. Thus, our design highlights the independence of the concept “hidden profile” of the specific case involving unshared items.

#### Metacognitive Monitoring and Correction

Because most collective learning is subject to selective repetition – due to unequal rates of majority and minority groups and variation in the information revealed by the environment – some arguments are more likely to be presented and repeated than others. But should it really be impossible to overcome this problem?

Taking a metacognitive perspective suggests an answer and a possible remedy. Because unequal sample sizes and repetition rates are ubiquitous in the real world, *homo sapiens* should have evolved meta-cognitive devices to monitor and correct for the impact of repetition. In the hidden-profile paradigm, selective repetition ought to be detected and correct for (e.g., B should be downward-corrected and D should be upward-corrected). From such a metacognitive theory perspective, it is not sufficient to point out that ordinary learning is sensitive to repetition; it is also necessary to explain why repetition and unequal validity are not corrected for.

The present approach relates an ordinary learning account to the intriguing notion of metacognitive myopia (Fiedler, 2000, 2012). Numerous findings demonstrate that sampling biases and repetition biases remain undetected and uncorrected at the metacognitive level (Fiedler et al., 2000, 2002, 2016; Unkelbach et al., 2007; Fiedler, 2012; Powell et al., 2017). For instance, Unkelbach et al. (2007) asked participants to assess how often 10 different shares were among the daily winners in a stock-market game. On some days, they watched two TV programs so that the winners were presented twice, creating a repetition bias in favor of these repeated daily winners. The chief determinant of the resulting evaluations and share preferences was the presentation frequency, regardless of whether presentations reflected new winning outcomes or mere repetitions. Strong and robust repetition bias persisted even when participants were deliberately warned to avoid being misled by mere repetitions.

Because of many similar findings in various paradigms (for a review, see Fiedler, 2012), we expected metacognitive-myopia to extend to hidden profiles. Learned preferences should be markedly biased, due to the failure to correct for apparent repetitions. Even explicit debriefing and warnings to ignore repetitions should not eliminate the bias. This expectation is easy to understand theoretically. One cannot tell one’s cognitive system to stop learning from repetitions (cf. Koriat, 1997; Fiedler et al., 2016; Powell et al., 2017), just as one cannot instruct oneself to stop learning from repeated CS-US pairings in Pavlovian conditioning.

Previous work on hidden profiles never mentioned the need for metacognitive monitoring and control, although metacognitive constructs were considered. Thus, Schulz-Hardt et al. (2016) assumed that discussion partners’ repetitions will reinforce the subjective validity rather than triggering an attempt to correct for repetition bias. Similarly, Weaver et al.’s (2007) notion that fluency mediates the evaluation of repeated arguments is suggestive of naïve and uncritical influences of metacognitive cues. The notion of metacognitive myopia is fundamentally different. We argue that a comprehensive account must not only explain why repetition biases (and feelings of fluency or social validity, and countless other biases) arise in the first place. It must also explain why repetition biases go undetected and uncorrected at the metacognitive level.

### Preview of Experiments and Predictions

For an empirical test of these considerations, we exposed individual participants to an audio-recorded protocol of verbal descriptions of positive and negative attributes of four target persons (A, B, C, and D). A cover story explained that targets were applicants for flat share and that the stimulus descriptions reflected the flat mates’ experiences with different subsets of applicants. To rule out group dynamics and social reward motives, participants were not engaged in group discussions but were individually exposed to a pooled (audiotaped) profile.

The four applicants varied in the *effective number* of positive versus negative attributes, such that the unequivocally correct preference order (D > C > B > A) should be apparent in a no-repetition baseline condition. However, by selectively repeating subsets of the targets’ positive and negative attributes (**Table 2**), the resulting *presentation frequencies* yielded a new ordering. This should cause a shift from the correct order D > C > B > A toward the repetition-based ordering B > D > A > C in Experiment 1. We expected that judges would fail to correct for repetition spontaneously. Even an explicit warning not to be misled by repetitions in one of two conditions should not undo the basic repetition effect on evaluative learning. Experiment 2 was devoted to another aspect of meta-cognitive myopia, namely, low sensitivity to variation in social validation. A repetition bias should be obtained regardless of whether repetitions came from the same source or from different flat mates (implying social validation).

In Experiment 3, the design was extended to include recall and recognition measures in addition to evaluative ratings, to substantiate the assumption that repetition fosters learning. To increase the reliability of memory tests, the number of items was doubled and four different patterns of target-item allocations served to enhance the external validity.

Moreover, Experiment 3 allowed for a more refined test of the meta-cognitive inability to correct one’s evaluative judgments. Instead of instructions not to learn from repetitions, which may be impossible, participants in one condition were informed that repetitions came from one flat mate who had vested interests in manipulating the decision. Such a cheater-detection prompt (Cosmides, 1989) entails an obvious demand to correct the final ratings of D relative to B. The vested-interest scenario should therefore motivate a local correction. However, the correction should not undo the impact of selective repetition on implicit learning, as evident in a persistent repetition bias in recall and recognition. Thus, despite the local correction of immediate ratings, the memory data may reveal that repetition biases have become an irreversible social reality.

## Experiment 1

### Methods

#### Participants and Design

Eighty-five participants (29 males and 56 females, mean age = 23.73, *SD* = 3.75) either received course credit or 3 Euro. One participant who did not complete the major dependent measures was excluded. The remaining 84 were randomly assigned to two instruction groups (warning vs. no warning). Another group of 15 participants received the same stimulus tape, from which all repetitions had been removed, to check on the premise that without repetitions the correct preference order (D > C > B > A) can be identified. Set sizes and numbers of positive and negative attributes per target (A, B, C, and D) varied within participants (**Table 2**).

In the absence of any effect size estimates from similar research, the number of participants required to meet a power criterion was hard to estimate. Given the rather high effect sizes obtained in Experiment 1, larger samples in Experiments 2 and 3 warranted overpowered tests, as evident from the evidence reported below.

#### Materials

In a pretest, 80 items describing positive (e.g., “He respects and pays heed to other people’s privacy,” “He always tries to preserve the harmony in the shared flat”) and negative attributes (e.g., “He is not very hospitable,” “He transfers a bad temper easily to his flat mates”) were rated by 26 judges for valence and importance for flat sharing. Two different stimulus series were constructed, such that the attributes of the four targets (cf. **Table 2**) were balanced for valence and importance. Only Series 1 was used in Experiment 1. Repetitions involved slightly altered but semantically invariant paraphrases of original items (e.g., “It is very hard to get him to help with the housework” repeated as “Getting him to help with the housework is very hard”). All items were presented vocally by three male volunteers; repetitions of the same items always came from different voices (flat mates). As all information about each target was presented as a randomly ordered block, repetitions were maximally detectable. Block order was counterbalanced.

#### Procedure

The entire experiment took place in computer dialog. Participants were asked to imagine living in a flat with four people, looking out for a new flat mate to replace one who had moved out. A casting would take place, during which applicants were interviewed by three flat mates. Not all of them were present when the applicants appeared, so the decision had to rely on a combined report of all flat mates’ experiences with subsets of applicants. One experimental group received an explicit warning not to be misled by repetition: “Some attributes of applicants may be stated repeatedly. Do not incorporate these repetitions in your evaluation.” This warning was not provided to the other group. Afterwards, participants rated the targets on five trait dimensions covering the meaning of the stimulus attributes (agreeable, communicative, appreciative, companionable, helpful; on graphical scales anchored “not at all” and “very much”). They also provided an overall evaluation of all candidates in response to the single item “How much would you like to share your flat with applicant X?”). All ratings were provided on graphical sliding scales; ratings were linearly transformed to numerical scores from 0 to 100. The entire experiment lasted between 10 and 15 min. The materials and computer procedures can be found under the following link: https://drive.google.com/drive/folders/1atdnNdyKAcdVhbWI6X-YOgg1itGpCkIQ?usp=sharing

### Results and Discussion

In accordance with the transparency norm, all empirical data are publicly available. To get access, click on *Hidden prof* on the site below: http://www.psychologie.uni-heidelberg.de/ae/crisp/studies/index.html

Average evaluation scores were computed across all five ratings. To make sure that in the absence of repetitions the stimulus attributes induced the intended ordering of targets (D > C > B > A), 30 participants provided baseline ratings of the targets in a questionnaire (using exactly the same rating scales and instructions as indicated above). Two subgroups evaluated targets described by two different versions of the stimulus series. These baseline ratings were also used to estimate the internal consistency of the five-item evaluation, which amounts to α = 0.91 when based on ratings averaged across all 30 judges, and α = 0.76 when the five ratings were used to discriminate between all 120 = 30 (judges) × 4 (targets) individualized targets. For convenience, we analyzed unweighted average ratings.^3^

#### Baseline Impressions

**FIGURE 1:**
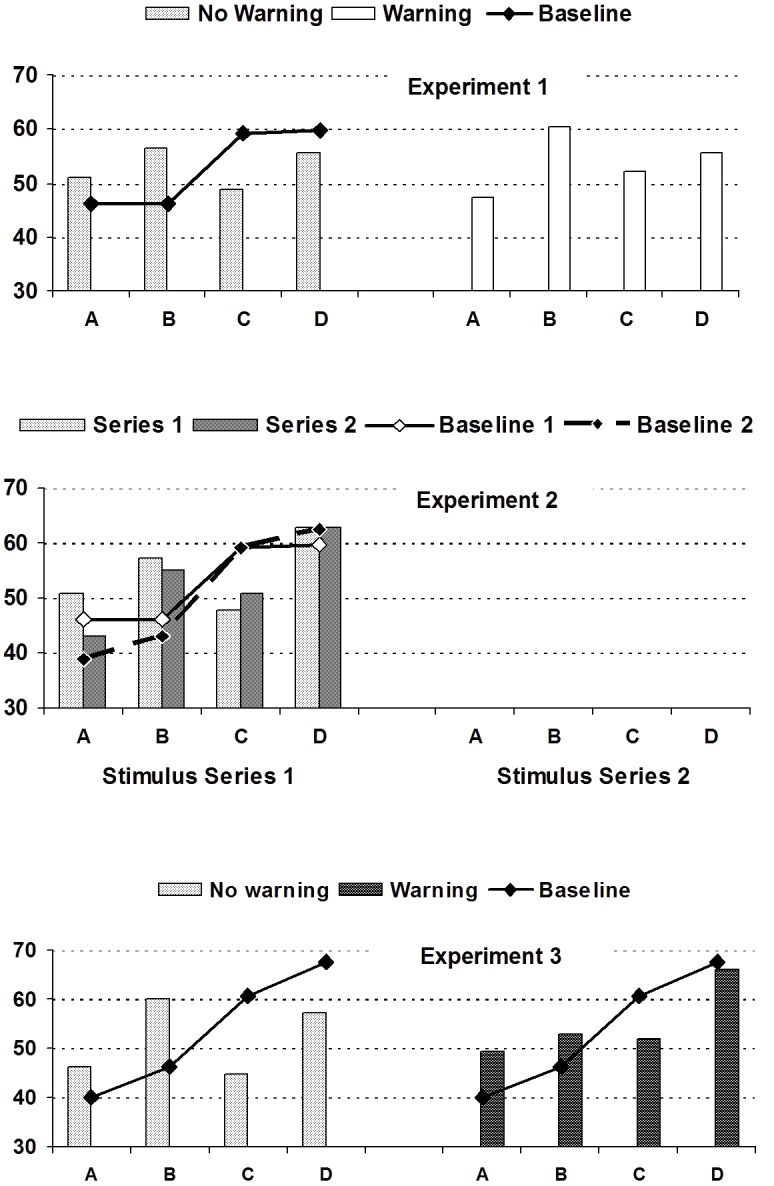
Mean evaluative ratings (averaged across traits) of target persons A, B, C, D by experimental conditions (warning vs. no warning vs. no-repetition baseline) across experiments.

Means and standard deviations of the baseline evaluation scores (without repetitions) are shown in **Table 3** (top row). Evidently, the stimulus series induced more positive impressions of the two superior targets (D,C) than the two inferior targets (B,A), although the two targets within each pair received similar ratings. While the four evaluation scores should have ideally produced a linear increase from A to D, the stepped line graphs in **Figure 1** suggest that the baseline evaluations were mainly sensitive to the difference between the two superior (D,C) and the two inferior targets (A,B).

**Table 3:** Means and standard deviations (italics) of target evaluations obtained in Experiments 1 and 2, as a function of instruction conditions (extra warning vs. no warning to ignore repetitions) and two stimulus series.

	Series 1				Series 2			
Target person	A	B	C	D	A	B	C	D
Baseline from pretest	46.23 *13.20*	46.18 *9.46*	59.10 *13.43*	59.78 *9.96*	38.92 *12.37*	43.32 *13.20*	59.69 *11.41*	62.41 *9.85*
Experiment 1 No warning	50.99 *12.27*	56.42 *13.92*	48.75 *12.11*	55.41 *13.72*				
Experiment 1 Warning	47.36 *11.00*	60.43 *11.45*	52.16 *10.45*	55.43 *12.72*				
Experiment 2 No warning	50.96 *15.84*	57.09 *13.44*	47.76 *13.93*	62.81 *10.47*	43.16 *11.70*	55.01 *13.84*	50.85 *9.80*	62.86 *11.61*

*Repetitions came from different speakers in Experiment 1 but from the same speaker in Experiment 2.*

For a statistical test of the intended baseline ordering, we followed Rosenthal and Rosnow’s (1985) advice to test focused hypotheses rather than standard analyses of variance, calculating a contrast score that captures a linear increase in evaluative ratings from A to D. This contrast score was the sum of each participant’s mean evaluation of A, B, C, and D, weighted by the *baseline contrast* coefficients -1.5, -0.5, +0.5, +1.5, respectively. Testing this baseline contrast against zero is tantamount to testing the discriminability of actually existing target differences, independent of repetitions. This premise was indeed met. The mean contrast score was clearly positive, *M* = +26.79, *SD* = 25.16 [CI 12.86; 40.72], *t*(14) = 4.12, *d* = 2.20, *p* = 0.001.

Note also that the sigmoid deviation from a purely linear trend (i.e., the slightly enhanced increase from B to C) cannot account for the repetition bias predicted for the experimental conditions (i.e., B > D > A > C), which implies that C should decrease markedly relative to B. This is evident from a *repetition contrast* defined as the sum of A, B, C, D evaluations weighted by the linear coefficients -0.5, +1.5, -1.5, +0.5, respectively, corresponding to the B > D > A > C pattern reflecting a repetition bias. Indeed, this contrast score tended to be negative, *M* = -12.60, *SD* = 25.32 [-26.62; 1.42], *t*(14) = -1.93, *d* = 1.03, *p* = 0.074, indicating that, if anything, the baseline evaluations worked against the predicted repetition bias.

A nice feature of the present design is that baseline and repetition contrasts are orthogonal; the cross product of -0.5, +1.5, -1.5, +0.5 and -1.5, -0.5, +0.5, +1.5 is exactly 0. This allows us to run independent tests of the impact of the effective number of positive and negative attributes (captured by the basic contrast) as well as the presentation frequencies (repetition contrast).

#### Repetition Bias on Target Evaluations

Turning to the experimental groups, the same average evaluation scores and contrast scores were used to analyze evaluations after selective repetition. As evident from the numerical means in the upper part of **Table 3** (summarized in **Figure 1**), the target evaluations reflect a mixture of both determinants, which is, however, clearly dominated by the repetition bias. Although the two superior targets C, D together received slightly higher evaluations than A, B, selective repetition caused a marked increase in the evaluation of A and B, along with a decrease in the evaluation of C and D, relative to the baseline. Explicit instructions to discount repetitions in the warning group did slightly decrease, but clearly not eliminate repetition biases (see **Figure 1**).

To disentangle the relative impact of the effective set size of different positive versus negative items and of the repetition bias, the baseline-contrast scores and the repetition-contrast scores were tested against zero. Across all 84 participants, the repetition contrast was strong and clearly above chance, *M* = +14.89, *SD* = 27.99 [+8.82; +20.96], *t*(83) = 4.874, *d* = 1.064, *p* < 0.001. The baseline contrast scores only slightly exceeded zero, *M* = +5.01, *SD* = 27.26 [-0.90; 10.93], *t*(83) = 1.684, *d* = 0.367, *p* = 0.096.

In the condition without an explicit warning to discount repeated items, only the repetition-contrast score was significantly positive *M* = +13.72, *SD* = 30.44 [4.88; 22.56], *t*(47) = 3.12, *d* = 0.91, *p* = 0.003, but not the baseline-contrast score *M* = +2.79, *SD* = 27.57 [-5.22; 10.80], *t*(47) = 0.70. This means that the repetition bias completely overrode the baseline evaluations.

An explicit warning to discount repetitions slightly increased the baseline-contrast score to a marginally significant level, *M* = +7.97, *SD* = 26.94 [-1.14; 17.08], *t*(35) = 1.78, *d* = 0.60, *p* = 0.084. However, the repetition-contrast score remained high and significant, despite the warning, *M* = +16.44, *SD* = 24.70 [8.08; 24.80], *t*(35) = 3.99, *d* = 1.35, *p* < 0.001. Indeed, the strength of repetition bias increased slightly after a warning (from 13.72 to 16.44). While this difference was far from being significant, *t*(82) = 0.44, *p* = 0.661, it highlights the ineffectiveness of the warning.

The single-item summary evaluation yielded a similar ordering as the overall evaluation score based on five trait ratings (*M* = 41.26, 51.48, 45.98, 49.62, *SD* = 23.78, 25.58, 24.72, 24.72, for A, B, C, and D, respectively). Due to the restricted reliability of this single-item measure, though, both the repetition-contrast, *M* = +12.42, *SD* = 58.01 [-0.17; 25.01], *t*(83) = 1.96, *d* = 0.43, *p* = 0.053, and the baseline contrast *M* = +9.78, *SD* = 51.37 [-1.37; 20.93], fell short of significance, *t*(83) = 1.75, *d* = 0.38, *p* = 0.084.

Altogether, these findings support the notion that even when all collective knowledge is shared, the resulting judgments are clearly biased. Mere repetitions of original items caused a marked bias in favor of A and B and against C and D, as portrayed in **Figure 1**. This finding fits a fully normal law of learning. As learning increases with repetitions, it is no wonder that the impact of repeated information on evaluations is enhanced. Yet, it is reflective of meta-cognitive myopia, the inability to correct for selective repetition.

However, as repetitions in Experiment 1 always came from different speakers, they may have been understood as social validation. Although this cannot account for the failure of explicit discounting instructions, it may have facilitated the repetition bias. To rule out this possibility, we conducted a new experiment with repetitions always coming from the same speaker. If meta-cognition is sensitive to social validation, the repetition bias should disappear, or the resulting judgments should be at least reduced relative to the different-speaker condition in Experiment 1. Conversely, if clearly redundant same-person repetitions continue to exert a systematic bias, this would lend further support to metacognitive myopia.

Another limitation of Experiment 1 was the constant assignment of attributes to targets. In Experiment 2, we used two different stimulus tapes (Series 1 and 2) with different assignments.

## Experiment 2

### Methods

#### Participants and Design

Fifty-four students (9 males and 45 females; mean age = 23.93, *SD* = 6.19) of Heidelberg University participated either for payment (3 Euro) or for course credit. The same distribution of positive and negative target attributes was used as in Experiment 1.

#### Materials

To rule out specific material effects, two different stimulus tapes with different assignments of specific attributes to targets (cf. **Table 2**) were assigned to different participants.

#### Procedure

All participants received instructions without a warning to discount repetitions. Unlike Experiment 1, all repetitions came from the same speaker, highlighted by the block-wise presentation of all items per target. Otherwise the procedure was identical to Experiment 1.

### Results and Discussion

#### Baseline Impressions

**Table 3** (right part) shows that both stimulus series led to very similar baseline evaluations, consistent with the intended ordering D > C > B > A. The mean baseline-contrast score for the new tape was highly positive, *M* = 43.43, *SD* = 27.52 [CI 28.19; 58.67], *t*(14) = 6.11, *d* = 3.27, *p* < 0.001. The mean repetition-contrast score was again negative, *M* = -12.81, *SD* = 22.99 [CI -25.54; -0.08], *t*(14) = -2.16, *d* = 1.15, *p* < 0.049. Any material bias should thus render the test of a repetition bias conservative.

#### Repetition Bias on Target Evaluations

Indeed, sensitivity to the effective differences in positive and negative target attributes was enhanced when repetitions came from the same speaker, thus ruling out any social-validation effect. Same-speaker repetitions apparently sensitized judges to the actual differences between targets. However, this did not eliminate or reduce the repetition bias. Though the baseline contrast score was elevated, *M* = +20.30, *SD* = 28.35 [12.56; 28.04], *t*(53) = 5.26, *d* = 1.45, *p* < 0.001, repetition contrast scores remained high and significant, *M* = +18.00, *SD* = 29.14 [10.05; 25.95], *t*(53) = 4.54, *d* = 1.25, *p* < 0.001. Both versions of the stimulus input replicated the same basic pattern (see **Table 3**).

A comparison of Experiments 1 and 2 (drawing on same materials and participant pool) corroborates the impression of similar repetition biases induced by different and same speakers, *M* = +14.89 vs. +18.00, *t*(136) = -0.628, *d* = -0.541, *p* = 0.531. Independent of this comparison across experiments, the strong and significant repetition bias obtained with same speakers in Experiment 2 highlights the metacognitive insensitivity to lack of social validation.

## Experiment 3

So far, we have silently assumed that the tenacity of the repetition bias reflects a natural learning advantage of repeated stimuli. For an empirical check on this assumption, the design of Experiment 3 was augmented to include a free recall test and a recognition test. Both memory measures were expected to reflect the learning advantage of repeated items. To render the two memory tests sufficiently reliable, the number of stimulus items used was doubled (**Table 2**, bottom part). Thus, Experiment 3 also affords an extended replication.

While an ordinary-learning approach clearly predicts that repetition biases should be manifested in memory performance, this need not imply that the repetition effect on target evaluations is mediated by its effect on memory. It is not clear whether the final target judgments are memory based or reflective of an online process of continuous updating taking place during stimulus presentation (Hastie and Park, 1986; Hogarth and Einhorn, 1992). Such an instance-based online learning process (cf. Gonzalez and Dutt, 2011) may not produce a strong correlation between item memory and evaluative judgments. People who produce the strongest repetition bias in target ratings need not also exhibit the strongest bias in target ratings. Selective repetition (R) might be a common cause of independent biases in memory (M) and judgment (J) tasks. Experiment 3 also offers an opportunity to compare such a common-cause model R → M,J against a mediation model R → M → J.

Furthermore, Experiment 3 included a new manipulation to gain a more refined picture of the meta-cognitive inability to correct for selective repetition. Assuming that people cannot undo repetition effects on learning does not mean that they cannot correct their final judgments on demand. When told that repetitions come from flat mates with vested interests in manipulating the target evaluations, judges may easily follow the demand and downgrade B and A (who profit from repetition) relative to C and D (who suffer from repetition). But such a demand-driven correction will hardly undo the learning-advantage of repetition. The bias should still be alive in recall and recognition, waiting to become social reality and to be utilized in future judgments and communications.

### Methods

#### Participants and Design

Ninety-five students of the University of Heidelberg (27 male, 68 female; average age 23.14, *SD* = 4.03) participated either for payment (6 Euro) or to meet a course requirement. They were randomly assigned to two experimental groups (warning vs. no warning) that only differed in whether or not instructions provided a warning to ignore selective repetitions from flat mates with vested interests. Recall and recognition tests were included along with the target ratings. A separate baseline group (*n* = 80) rated the four targets based on one of four new stimulus series without repetitions.

#### Materials and Procedures

The same materials and procedures were used as in all previous experiments, except for three distinct changes. First, the number of stimulus attributes was doubled to base the memory tests on a reasonable number of items. As shown in **Table 2**, the number of attributes was now 12 for targets A, B, and C and 16 for target D. The presentation frequencies resulting from selective repetition were also twice as high as in Experiments 1 and 2.

The selection and pre-scaling of the enlarged stimulus set were accomplished in a new pilot study, in which 28 judges rated 114 attributes relevant to flat sharing for valence. Four different versions of the stimulus series were constructed, balanced for importance of positive (e.g., “It is important for him that he has a good relationship with his roommates,” “He takes care and respects the inventory in the flat”) and negative behaviors associated with the four targets (e.g., “He is not very dependable”). All 80 items (52 basic attributes plus 28 repetitions; cf. **Table 2**) were tape-recorded and presented vocally by three male volunteers.

In the warning condition, all positive repetitions of target B and all negative repetitions of C and D always came from the same voice (one for each target), consistent with the suggestion that someone had vested interests in upgrading or downgrading one particular target. Repetitions of target A attributes came from all three voices. According to the instructions, target A was known by all speakers because they had recently met him at a birthday party. Target B was said to be a study mate of one speaker, who was therefore interested in B’s help on home work and exam preparation. C was said to be unwanted by another speaker, because they both owned a car and they would have to compete for a single parking slot. The reason for the third speaker to avoid target D was that bathroom conflicts could be anticipated because both had to get up and rush to work early in the morning. Pragmatically, then, it was easy to see that B ratings ought to be downward-corrected whereas C and D ought to be upward-corrected.

In the no-warning condition, the stimulus series consisted of four counterbalanced blocks of target descriptions presented by the same speaker (and thereby minimizing social validation).

Two computerized memory tests were presented at the end of the session. The recall test always preceded the recognition test. Participants were asked to write down all attributes they could recall in separate text fields for targets “A,” “B,” “C,” and “D” (presented in random order). Responses were scored as correct if they reflected the correct target reference and the substance of an original item, according to two independent coders who were blind for conditions (Cohen’s Kappa = 0.92). Separate recall proportions were calculated for singular and repeated attributes (pooling double and triple repetitions).

The final 71-item recognition test consisted of all 52 original items intermixed with 19 new items that had been never presented. Items were presented on head phones in random order. Participants were then asked on screen, without time constraints, whether the prompted item had been included in the list. If the answer was “Yes,” they had to indicate the target with which the item had been associated. We also assessed the confidence of recognition responses but refrained from analyzing these data. Two separate measures were calculated, the proportion of correctly recognized unrepeated items and the corresponding recognition proportion for repeated items.

### Results and Discussion

#### Baseline Impressions

We first of all conducted a test of the premise that, in the absence of repetitions, the effective number of targets’ positive and negative attributes would produce the ordering *D* > *C* > *B* > *A*. The mean evaluation scores clearly increased as intended from A to D (see **Table 4** and solid lines in **Figure 1**). Closer analyses revealed that this premise held for all four versions of the stimulus tape. For an empirical check, we computed the same baseline contrast scores as in previous experiments, summing up the evaluation scores of targets A, B, C, and D weighted by the contrast coefficients -1.5, -0.5, +0.5, +1.5. The average baseline contrast score in the baseline condition was highly positive, *M* = +49.89, *SD* = 30.85 [43.03; 56.75] and different from zero, *t*(79) = 14.46, *d* = 3.25, *p* < 0.001.

**Table 4:** Means and standard deviations (italics) of target evaluations obtained in Experiments 3 as a function of instruction conditions (warning vs. no warning).

	No warning				Warning			
Target person	A	B	C	D	A	B	C	D
Baseline (no repetition)	39.93 *12.99*	46.17 *12.11*	60.59 *11.05*	67.59 *11.87*			
Version 1	43.07	50.20	62.99	65.73			
Version 2	35.37	50.28	58.50	69.47	Same baseline data hold for the no-warning and the warning condition		
Version 3	39.26	39.72	57.57	65.44			
Version 4	39.84	42.16	61.96	70.64			
Target evaluations	46.39	60.03	44.78	57.31	49.48	52.76	51.88	65.99
	*13.21*	*14.20*	*12.39*	*12.62*	*13.22*	*15.36*	*12.07*	*14.24*

Again, we also computed the repetition-contrast scores to rule out the possibility that the expected repetition bias in the experimental conditions may be peculiar to specific stimuli. Contrary to such a bias, the repetition contrast score (i.e., A, B, C, D ratings weighted by coefficients -0.5, +1.5, -1.5, +0.5) actually tended to take on a negative value, *M* = -7.71, *SD* = 24.54 [-13.17; -2.25], *t*(79) = -2.81, *d* = 0.63, *p* > 0.001. Thus, as in Experiments 1 and 2, the baseline impressions were slightly working against the experimental prediction; a contrast capturing the repetition pattern B > D > A > C tended to be negative.

#### Repetition Bias on Target Evaluations

Despite this conservative bias in the stimulus materials, the introduction of selective-repetitions caused a strong shift toward positive repetition-contrast scores. Across both conditions, the distribution of repetition contrast scores was clearly above zero, reflecting the expected repetition bias, *M* = +18.45, *SD* = 28.50 [12.64; 24.26], *t*(94) = 6.310, *d* = 1.295, *p* < 0.001. The baseline-contrast score was also significant across both conditions, *M* = +15.77, *SD* = 34.46 [8.75; 22.79], *t*(94) = 4.461, *d* = 0.915, *p* < 0.001.

The strength of the repetition bias, however, was moderated by the warning manipulation. When participants did not receive a warning that selective repetitions came from speakers with vested interests, the repetition-contrast score was positive and highly significant, *M* = +28.34, *SD* = 25.82 [20.76; 35.92], *t*(46) = 7.60, *d* = 2.22, *p* < 0.001. As in previous experiments, a marked repetition bias was manifested in elevated ratings of target B but lower evaluations of target D (see **Table 4** and **Figure 1**). The baseline-contrast score fell short of significance, *M* = +7.90, *SD* = 32.34 [-1.60; 17.40], *t*(46) = 1.691, *d* = 0.493, *p* = 0.098. Apparently, then, judgments were no more sensitive to independent attributes than to repetitions.

However, in the warning condition, the repetition bias was greatly reduced, though not fully eliminated. The mean repetition-contrast score, *M* = +9.56, *SD* = 28.55 [1.27; 17.85], was still positive, *t*(47) = 2.35, *d* = 0.68, *p* = 0.023, though clearly lower than in the no-warning group (cf. **Table 4**), *t*(93) = 3.39, *d* = 0.70, *p* = 0.001.^4^

The effect of warning conditions testifies to judges’ ability to modify their ratings in accordance with explicit hints to deceptive behavior (bottom chart of **Figure 1**). At the same time, the blatant warning served to strengthen the original (baseline) ordering, as evident in positive and significant baseline-contrast scores, *M* = +24.34, *SD* = 35.04 [14.16; 34.52], *t*(47) = 4.86, *d* = 1.40, *p* < 0.001, which were higher than the baseline contrast scores in the no-warning condition, *M* = 7.90, *SD* = 32.34, *t*(93) = 2.227, *d* = 0.459, *p* = 0.028.

Apparently, then, when participants know that selective repetitions serve a manipulative goal, they are capable of correcting their final ratings. If speakers have vested interests in benefitting B and harming C and D, judges know how to correct for the bias: one only has to downgrade B ratings and to upgrade C and D ratings. The crucial question, though, is whether this correction eliminates the mental extract of the repetition bias or whether it merely changes the overt judgment output. The correction might remain superficial while the repetition bias might live on in the judges’ memory, waiting to influence later judgments or actions. Both memory measures afford a straightforward test of this challenging issue. Even though participants were apparently able to correct for a bias on overt rating scales, they may not be able to undo the uncontrollable effect of stimulus repetition on recall and recognition.

#### Recall

Indeed, across all participants, the correct-recall proportion of repeated items was much higher, *M* = 0.120, *SD* = 0.100 [CI 0.109; 0.150], than proportions of recalled unrepeated items, *M* = 0.059, *SD* = 0.054 [CI 0.048; 0.070], *t*(94) = 6.83, *d* = 1.39, *p* < 0.001. This recall advantage of repeated items was similarly strong in the no-warning condition, *M* = 0.126, *SD* = 0.104 [CI 0.096; 0.156], versus *M* = 0.064, *SD* = 0.057 [CI 0.047; 0.081], *t*(47) = 4.95, *d* = 1.44, *p* < 0.001, as in the warning condition, *M* = 0.113, *SD* = 0.097 [CI 0.085; 0.141], versus *M* = 0.054, *SD* = 0.051 [CI 0.039; 0.069], *t*(48) = 4.66, *d* = 1.35, *p* < 0.001. Thus, the blatant warning did not reduce the strength of the repetition bias in recall, regardless of the corrections applied to the immediate target ratings.

#### Recognition

The analysis of the recognition data provided further support for the persistence of the repetition bias, although the pattern was not quite the same. Across all participants, responses on the combined recognition and assignment test were more likely to be correct for repeated items, *M* = 0.372, *SD* = 0.145 [CI 0.342; 0.401], than for unrepeated items, *M* = 0.324, *SD* = 0.102 [CI 0.302; 0.346], *t*(94) = 2.91, *d* = 0.60, *p* = 0.005. However, notably, this tendency was not significant in the no-warning condition, *M* = 0.361, *SD* = 0.155 [CI 0.315; 0.406], versus *M* = 0.327, *SD* = 0.100 [CI 0.298; 0.356], *t*(46) = 1.53, *d* = 0.45, *p* = 0.132. Ironically, it was stronger in the warning condition, *M* = 0.382, *SD* = 0.135 [CI 0.343; 0.422], versus *M* = 0.321, *SD* = 0.115 [CI 0.288; 0.354], *t*(47) = 2.52, *d* = 0.74, *p* = 0.015.

Thus, although the repetition bias was somewhat weaker in recognition than in recall, the evidence from both memory tests supports the notion that the bias persisted in memory although deliberate responses on rating scales could be corrected in accordance with instruction demands. Even when stimulus input is strongly discredited, metacognition can hardly tell the cognitive system not to learn from repetition. An interesting question for future research is whether the memory advantage of repeated information also persists after a longer delay.

#### Relating Judgment Biases to Memory Measures

Finally, it is interesting to examine the relationship between individual judges’ repetition contrast scores and their corresponding biases in the two memory tasks. In fact, both correlations turned out to be low. Individual differences in the repetition contrast scores were only weakly correlated with individual measures of the differential proportions of correctly recalled repeated items minus correctly recalled singular items, *r*(*df* = 93) = 0.152, *p* = 0.142. When computed separately per condition, this correlation was close to zero without a warning, *r*(*df* = 45) = 0.063, *p* = 0.676, and slightly higher after a warning, *r*(*df* = 46) = 0.230, *p* = 0.116. The corresponding correlations between repetition contrast scores and differential recognition proportions for repeated minus singular items were negligible: *r*(*df* = 93) = -0.051, *p* = 0.625 across all participants; *r*(*df* = 45) = -0.016, *p* = 0.917 without a warning, and *r*(*df* = 46) = -0.029, *p* = 0.845 after a warning.

Relying on a total of 95 participants, these small correlations can be hardly attributed to insufficient statistical power. Although the present experiments were not designed to allow for strict tests of the underlying mechanism, the range of correlations is hardly compatible with the assumption that evaluative biases are substantially mediated by selective memory biases. Much more likely than a memory-based judgment process is the assumption that evaluations are learned online (Hastie and Park, 1986) and that evaluative ratings and memory responses are influenced by the same common cause.

## General Discussion

The hidden-profile paradigm continues to fascinate scientists; it is at the heart of democratic culture. Democracies deputize decisions to collectives relying on a division of labor, calling for the integration of the knowledge and expertise of several agents or advisors. However, the available evidence (Kerr and Tindale, 2004) shows that people have a hard time to coordinate and exploit collective knowledge. Three decades of illuminating experimental research in the hidden-profile paradigm testify to this problem.

The failure to solve hidden profiles has been explained in terms of such group-dynamic factors as the reward and the social validation value of shared information (Wittenbaum et al., 1999; Greitemeyer and Schulz-Hardt, 2003), the memory advantage of shared over unshared arguments (Lightle et al., 2009), and decision schemes favoring arguments consistent with pre-existing individual preferences (Edwards and Smith, 1996; Schulz-Hardt et al., 2016). Prior research has also noted that shared and preference-consistent arguments are likely to be repeated and that repetition (Schulz-Hardt et al., 2006; Stasser et al., 2012) and resulting feelings of fluency (Weaver et al., 2007) can influence subsequent target judgments.

However, prior research and theorizing never elaborated on the fundamental rule that all evaluative learning increases with the number of trials and that frequency of presentation and repetition are therefore primary causal variables that can explain troubles with hidden profiles independently of motivated biases and group-dynamics. Even when all items are shared and when there is no extra motive to process particular items more than others, the presentation rate of different information items will always vary as a function of many environmental conditions. In the absence of any bias to attend to or to elaborate on specific arguments more than on others, presentation rates will be higher for majority than minority arguments, for proximal than distal events, for ingroups than outgroups, and for public than private knowledge, to list but a few ecological determinants of item frequency.

From such an ordinary-learning perspective, information sharing, preference consistency, and social validation are only special cases of a much broader class of environmental causes of selective presentation and repetition. Even when all the prominent factors emphasized in previous research are controlled for, one cannot expect the problem with hidden profiles to be resolved because other, often quite normal, factors will continue to create selective repetition. Some daily news are encountered twice or more often; sometimes “breaking news” is indeed recycled abundantly; the same emails often reach us multiple times; misunderstandings or debates may motivate argument repetitions, or in democratic discussions, the same points are stated more frequently if held by a majority (Fiedler and Wänke, 2009). And last not least, in science, frequently cited findings have a clear-cut repetition advantage.

Because repetition biases are ubiquitous and because it is impossible not to learn from repetition, a major role is assigned to metacognition. If repetition biases are unavoidable in the first place, because the opportunity to learn is never the same across all items, monitoring and control functions are required to detect and correct for repetition biases. However, the present findings demonstrate that participants fail to correct for selective repetition. Although the blocked presentation mode facilitated the detection of repetitions, and when repetitions coming by the same speaker minimized their social validation value, evaluative judgments continued to be biased toward selective repetitions. Moreover, even when judges were sensitized through explicit debriefing and instructions not to be misled by selective repetitions, ruling out pragmatic demands to consider repeated arguments valid, the repetition bias could not be erased.

Note that the causal role assigned to metacognitive monitoring and control is independent of whether repetition rates might be correlated with other factors such as fluency, inferred consensus, or subjective validity. The theoretical importance of metacognition is independent of such natural confounds of repetition frequency. Regardless of what experiential cues drive the enhanced impact of repeated arguments – fluency, social validity, or sharedness – the correction of unavoidable repetition biases calls for metacognitive monitoring and control functions.

It is not too surprising, of course, that metacognitive control cannot undo automatic learning. One cannot tell the cognitive system to cease learning from repetition, just as we cannot tell our body to cease learning from repeated pairing of conditional and unconditional stimuli in Pavlovian conditioning. Unsurprising as this contention may be, it has distinct and memorable implications for collective judgments and decisions in democratic societies. Simply allowing every argument to be presented is no guarantee for unbiased judgments and decisions. Rarely presented minority arguments will more likely be ignored, forgotten or overridden than frequently presented majority arguments. To correct for this inevitable learning asymmetry, it would be necessary to allow rare arguments or minority positions to be presented more often than common majority positions. However, such an ironic minority privilege would not be compatible with the spirit of democracy either. Democratic rules alone cannot solve the dilemma. Rather, the burden of rational decision making rests on democratic agents’ meta-cognitive ability to distinguish valid from invalid, original arguments from redundant repetitions.

The present findings strongly suggest that metacognitive myopia prevents homo sapiens from this kind of critical assessment, adding convergent evidence to existing findings on metacognitive myopia (Fiedler, 2000, 2012; Fiedler et al., 2016).

We anticipate that it will hardly be possible to prevent the initial occurrence of repetition biases in the first place. We rather believe that the existence of this ubiquitous source of bias must be taken for granted as a natural product of environmental learning. It can only be diagnosed and corrected at the metacognitive level. However, a host of convergent evidence suggests that metacognitive myopia prevents homo sapiens from critical assessment and correction (Fiedler, 2000, 2012; Fiedler et al., 2016). Even explicit reminders not to be misled by selective repetition and lopsided sampling do not prevent people from adopting the more frequently presented arguments. The present findings corroborate this conclusion in the context of collective judgments: even when social validation is ruled out and when blocked presentation makes argument repetition maximally visible, and sometimes even after a warning to avoid a repetition bias, participants continue to be strongly influenced by mere repetition.

Note that metacognitive myopia affords a functionalist account rather than a mechanistic (Fiedler, 2016). It highlights the failure to engage in metacognitive monitoring and control functions, which might involve a variety of different mental algorithms. For some reason, *homo sapiens* is not sufficiently motivated or may have actively learned not to engage in retrograde correction of even blatant sampling biases (Fiedler, 2008, 2012). We exhibit perseverance after full debriefing that some feedback was completely wrong (Ross et al., 1975); we continue to be influenced by fake news after debunking (Chan et al., 2017), we treat advertising as a source of evidence and citation rates as a symptom of good science, without any attempt to control for obvious sampling biases.

One may speculate that metacognitive myopia serves adaptive functions, conserving one’s faith in the validity of the empirical world and preventing people from tedious correction processes for which there is often no normative solution. Alternatively, there may have been insufficient selection pressure, maybe because metacognitive monitoring and control has only lately become important during a rather short information era, or it may simply not constitute a genuine survival advantage. Nevertheless, in the context of specific problems, such as personnel selection or investment decisions, it would be beneficial to develop decision aids and training programs to overcome the constraints of metacognitive myopia, to avoid injustice and irrational action.

## Ethics Statement

This study was carried out in accordance with the recommendations of the German Association of Psychology (DGPs). All subjects gave written informed consent in accordance with the Declaration of Helsinki.

## Author Contributions

All authors listed have made a substantial, direct and intellectual contribution to the work, and approved it for publication.

## Conflict of Interest Statement

The authors declare that the research was conducted in the absence of any commercial or financial relationships that could be construed as a potential conflict of interest.

## Footnotes

1 A corollary of this account is that memory overload can increase redundancy gains (Tindale and Sheffey, 2002).

2 Schulz-Hardt et al. (2016) used only two options that only differed in repetitions.

3 Fully equivalent results were obtained (in all pilot tests and experiments) when the five traits were weighted proportionally to their rated relevance to flat sharing (i.e., 0.228, 0.178, 0.191, 0.216, and 0.187, respectively, for agreeable, communicative, appreciative, companionable, and helpful), as determined in further pilot testing.

4 The only significant result in a 2 (warning conditions) × 4 (material versions) ANOVA was a main effect for conditions, *F*(1,87) = 11.181, *p* = 0.001. Neither the material versions main effect, *F*(3,87) = 1.775, *p* = 0.158, nor the interaction was significant, *F*(3,87) = 0.402, *p* = 0.752, reflecting a robust effect that is not peculiar to specific stimuli.
